# Hemorrhagic Posterior Reversible Encephalopathy Syndrome in a Pediatric Patient With Acute Lymphoblastic Leukemia: A Case Report

**DOI:** 10.7759/cureus.57158

**Published:** 2024-03-29

**Authors:** Kristīne Kalēja, Artūrs Sokolovskis, Inga Ziemele

**Affiliations:** 1 Faculty of Medicine, Riga Stradins University, Riga, LVA; 2 Department of Pediatrics, Faculty of Medicine, Riga Stradins University, Riga, LVA; 3 Department of Pediatric Infectious Diseases, Children’s Clinical University Hospital, Riga, LVA

**Keywords:** hypertension, chemotherapy, intracerebral hemorrhage, acute lymphoblastic leukemia (all), posterior reversible encephalopathy syndrome (pres)

## Abstract

Posterior reversible encephalopathy syndrome (PRES) is an uncommon yet severe neurological disorder characterized by a combination of clinical and radiological features. Common clinical presentations of PRES include headaches, seizures, altered mental status ranging from lethargy to coma, visual disturbances, and behavior changes.

This case report outlines the occurrence of hemorrhagic PRES in an 11-year-old girl with B-cell acute lymphoblastic leukemia (ALL) relapse. Hospitalized for ALL relapse, the patient underwent reinduction chemotherapy. On the ninth day of admission, she had a generalized tonic-clonic seizure with a blood pressure peak of 170/120 mmHg. Magnetic resonance imaging (MRI) and a seizure episode suggested PRES. Initially, after the first tonic-clonic seizure, the neurological examination was normal, but after the second seizure, the meningeal symptoms were negative, and gaze palsy and right-sided homonymous hemianopsia were observed; muscle strength was symmetrically reduced in the upper and lower extremities and reflexes were symmetrical and diminished. A bilateral Babinski reflex was observed at the time of examination; the patient had mild motor aphasia, and she opened her eyes only in response to tactile stimulation. A follow-up MRI four days after the second seizure episode showed extensive PRES damage with hemorrhagic changes. Over two weeks, the patient's neurological status and blood pressure gradually improved, with persistent changes in the visual field. Subsequent MRI revealed a significant reduction in PRES lesions, but residual hemorrhage measuring 6x4 cm remained evident.

## Introduction

Posterior reversible encephalopathy syndrome (PRES) is a neurological disorder that was first recognized in 1996 by Hinchey and colleagues, characterized by a combination of clinical symptoms and radiological features [1]. It is characterized by the rapid onset of various neurological signs and symptoms, including seizures, altered mental status, focal neurological deficits, headaches, vomiting, and visual disturbances. While the exact mechanisms behind PRES remain unclear, it has been observed in children in connection with various conditions, the most frequent being hypertension, exposure to cytotoxic substances, systemic lupus erythematosus, and renal disorders [2,3]. The diagnosis is confirmed by neuroimaging, which typically shows symmetrically distributed areas of vasogenic edema, mainly within the posterior circulation territories [4]. Complete recovery is usually achieved by supportive care and withdrawal of the offending agent, supplemented by the addition of antiepileptic and antihypertensive therapy if needed [5].

In this article, we describe the case of a pediatric patient who developed PRES with secondary intracerebral hemorrhages (ICH) associated with hypertension and cytotoxic drug exposure during reinduction chemotherapy for acute lymphoblastic leukemia (ALL).

## Case presentation

We present the case of an 11-year-old girl with B-cell ALL with a high-risk (HR) translocation t(17;19) who developed hemorrhagic PRES during reinduction chemotherapy. She was diagnosed with ALL in March 2023.

In September 2023, the patient was admitted to the hematology/oncology department to undergo reinduction chemotherapy according to the IntReALL 2010 protocol due to HR combined relapse involving both the central nervous system and extramedullary regions, with the emergence of new extramedullary lesions in the mandible and the right parietal bone. On the day of admission, her overall condition was characterized as moderately severe, and the physical examination revealed intact neurological status with a Glasgow Coma Scale (GCS) score of 15, isochoric and light reactive pupils, and no meningeal signs or focal deficits. Elevated blood pressure of 125/90 mmHg (99th percentile + 12 mmHg) was noted, and antihypertensive therapy was initiated. In the following days of hospitalization, she received reinduction chemotherapy consisting of vincristine (1.69 mg) and methotrexate intrathecally (12 mg), a single dose of each.

**Figure 1:**
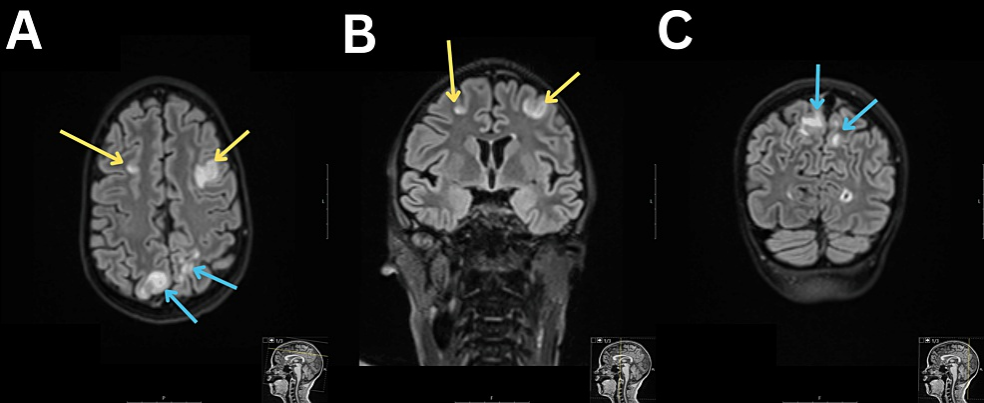
T2-Weighted MRI MRI FLAIR images showing hyperintense lesions in cortical and subcortical white matter in the frontal (A and B, yellow arrows) and parietal regions (A and C, blue arrows). MRI, magnetic resonance imaging; FLAIR, fluid-attenuated inversion recovery.

On the ninth day of hospitalization, the patient developed a generalized tonic-clonic seizure episode with uprolling of the eyes and loss of consciousness. For seizure control, she received diazepam, midazolam, and valproate. During the seizure episode, the patient's blood pressure was 170/120 mmHg, and the hypertensive state was managed with combined antihypertensive therapy. Magnetic resonance imaging (MRI) of the brain showed focal, cortical, and subcortical vasogenic edema in both frontal and parietal lobes, suggesting PRES (Figure 1A-1C). Six days prior to this episode, an MRI was already performed on the patient due to the underlying illness, where such changes were not observed. The neurological examination findings after the seizure were as follows: the girl was oriented in time, place, and person with a GCS score of 15, the meningeal signs were negative, eye movements were full in range and no nystagmus was observed, the pupils were symmetric, and the reaction to light was preserved, no weakness in the arms and legs was observed, no sensory disturbances were observed, and the deep tendon reflexes of the legs were symmetric and they were rated as +1. The patient denied diplopia. Therapy with intravenous sodium valproate (35 mg/kg/day) was initiated. On the following day, the patient experienced another seizure episode, and she was transferred to the intensive care unit. During the night, a delirious episode began, characterized by confusion and visual and auditory hallucinations. 

The next day, during the morning examination, the patient appeared drowsy, had a vomiting episode, opened her eyes only in response to tactile stimuli, and showed signs of mild motor aphasia. Neurologically, the GCS score was 13, and the face was symmetrical, but partial gaze palsy to the right was observed. When examining the visual fields, there was an impression of right-sided homonymous hemianopsia. The range of motion in the extremities was sufficient, but there was a decrease in muscle strength. A bilaterally positive Babinski reflex was noted. An electroencephalogram (EEG) was performed, revealing focal epileptiform activity in the parieto-occipital regions, more prominent on the right side. The patient's blood pressure still remained within the range of 115-125/70-94 mmHg despite combined antihypertensive therapy with enalapril, nifedipine, labetalol, clonidine, furosemide, and spironolactone.

**Figure 2:**
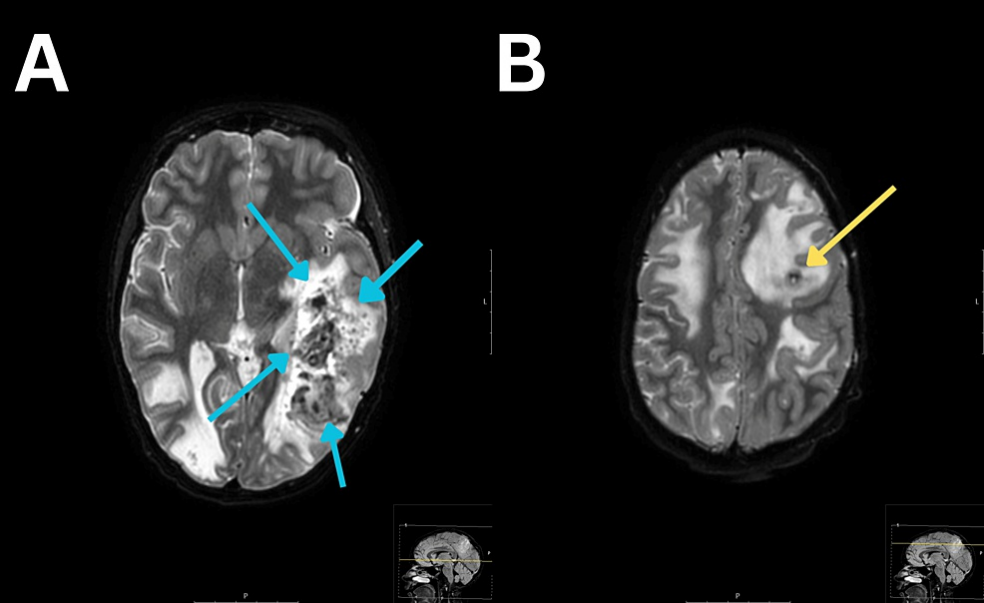
T2-Weighted MRI The blue arrows indicate a hemorrhagic imbibition in the left occipitotemporal region (image A). The yellow arrow shows a smaller hemorrhagic imbibition in the left frontal region (image B). MRI, magnetic resonance imaging.

Four days after the last seizure episode, a follow-up MRI of the brain was performed, which showed extensive PRES damage with widespread edema in the large hemispheres and a midline shift of the brain. There were also focal lesions in the cerebellum, and extensive hemorrhagic imbibition was observed in the left temporal and occipital lobes as well as in the left frontal lobe (Figure 2A, 2B). Despite significant deterioration in comparison to the previous MRI, with hemorrhagic changes being found, the condition of the patient was stable; she communicated slowly but adequately, appeared more alert, was physically more active, and responded to questions slightly more quickly, but still experienced difficulties finding words.

**Figure 3:**
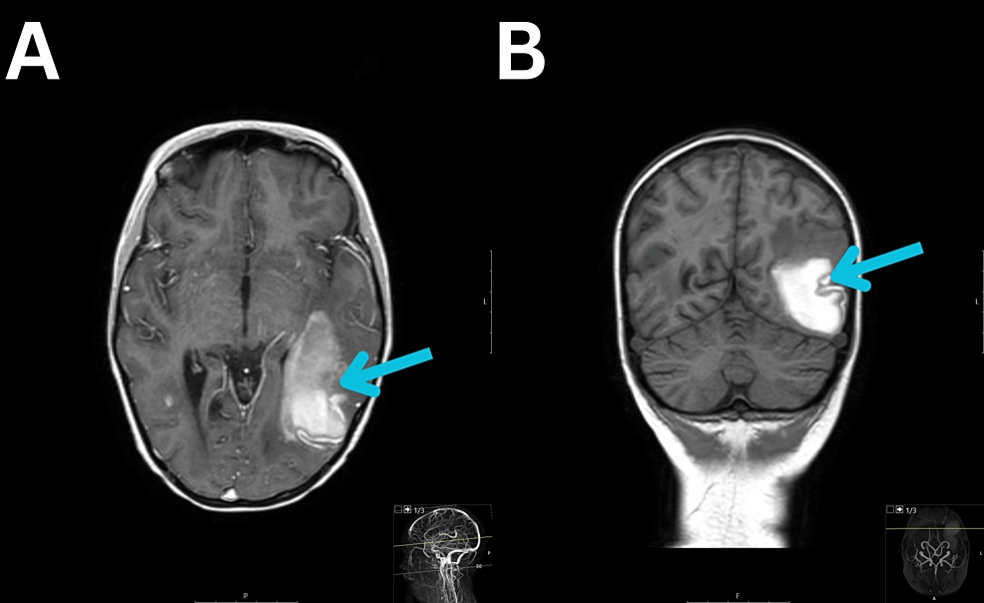
T1-Weighted MRI With and Without Contrast Intracerebral hemorrhage in the left occipitotemporal region (images A and B, blue arrows). MRI, magnetic resonance imaging.

After two weeks, her neurological condition improved, and she answered questions fluently, was oriented in time, place, and person, and obeyed commands. The muscle strength had returned to normal. Changes in the visual fields were still observed. The patient's blood pressure had normalized (above the fifth percentile and below the 50th percentile), and she was receiving daily therapy with enalapril and nifedipine. The control MRI showed a significant reduction in PRES lesions in the large hemispheres, with hemosiderin deposits and extensive hemorrhage in the left temporal and occipital lobes, measuring 6x4 cm (Figure 3A, 3B). The antiepileptic therapy was switched from intravenous to oral, and the patient continued to receive oral sodium valproate with a dosage of 1050 mg per day (33 mg/kg/day), divided into three doses.

One month after the initial seizure episode, a follow-up MRI examination revealed multiple deposits of hemosiderin indicative of past hemorrhages in the large hemispheres, lysis of the hematoma in the left occipital and temporal lobes, along with gliosis alterations in the parietal and occipital lobes. The patient did not report any new neurological complaints, and visual field disturbances had also resolved.

## Discussion

The exact pathophysiology of PRES is not fully understood; however, there are various proposed mechanisms to explain its occurrence. The first theory proposes that elevated arterial pressure disrupts the autoregulation of cerebral vessel pressure. This disruption subsequently results in cerebral hyperperfusion, breakdown of the blood-brain barrier, and damage to endothelial tight junctions, ultimately causing vasogenic edema. Therefore, according to this theory, there needs to be a sudden elevation in systemic blood pressure for PRES to occur. The second theory suggests that cytotoxic drugs, including chemotherapy agents used in our case report, can induce PRES through direct cytotoxic effects on the cerebrovascular endothelium, leading to the breakdown of the blood-brain barrier and vasogenic edema [5-9]. Our patient developed PRES, likely due to a combination of factors, including significant hypertension and exposure to induction chemotherapy. Chemotherapy was continued even after the onset of PRES symptoms because of the therapy-resistant ALL relapse, and it was not possible to rule out that the neurological symptoms were related to the progression of leukemia. She received mitoxantrone (5.6 mg) on the ninth and 10th days of hospitalization, followed by dexamethasone (7.5 mg) for five days.

The diagnosis of PRES relies on a combination of clinical and radiological findings. In the study conducted by Li et al., 556 patients were analyzed - both adults and children (median age: 34). The most prevalent clinical symptoms among them were headache (50.7%), altered mental status (43.7%), seizures (41.9%), visual disturbances (34.9%), nausea/vomiting (23.4%), and focal neurological deficits (18.2%) [10]. In another study done by Cordelli et al., where they analyzed 111 pediatric patients, results showed that 94.6% of patients presented with seizures, 23.4% had mental status changes not related to epileptic seizures, 12.6% presented visual disturbances, and only 6.3% reported headaches [2]. Our patient exhibited all the previously described symptoms, except for headaches.

Neuroimaging is crucial to exclude other possible differential diagnoses and to confirm PRES. Vasogenic edema, primarily affecting the posterior white matter of the cerebral hemispheres, is the typical neuroimaging manifestation of PRES. While vasogenic edema may be detectable with non-contrast CT in certain cases, MRI of the brain, especially T2-weighted sequences like fluid-attenuated inversion recovery, demonstrates significantly higher sensitivity. Typically, vasogenic edema is characterized by iso or hypointensity in diffusion-weighted imaging and hyperintensity in the apparent diffusion coefficient map [7,11].

Despite its name, PRES may manifest in locations other than the posterior distribution, occurring in areas such as the frontal, inferior temporal, cerebellar, thalamus, brainstem regions, and the spinal cord without cerebral hemispheric involvement [11-14]. In the research done in 2014, Donmez et al. reported that in pediatric patients frontal lobe involvement was almost as frequent as the occipital lobe (65.5%), and cerebellar involvement was also common: almost 50% of the patients they studied had cerebellar lesions [15]. In our patient, lesions were observed in the cerebellum, as well as in both frontal and parietal lobes, and the patient also developed ICH. In the study by Donmez et al. (2010), conducted with 33 patients (ranging from six years to 69 years old; mean age: 25 years), 27% experienced the complication of hemorrhage [16]. However, in a 2014 study that aimed to compare MRI observations between adult and pediatric patients with PRES, four out of 29 (14%) pediatric patients were found to have hemorrhage on MRI, suggesting that hemorrhagic complications in the pediatric population could occur at almost the same frequency as in adults [15].

The management of PRES involves supportive care and addressing the underlying cause, supplemented by the addition of antiepileptic and antihypertensive therapy if needed [5]. Patients should receive adequate hydration, and any imbalances in electrolytes should be addressed. Those experiencing increased intracranial pressure due to cerebral edema may necessitate neurosurgical interventions [17].

## Conclusions

In conclusion, it is important to consider PRES as a differential diagnosis for patients who experience symptoms like seizures, headaches, altered mental status, and focal neurological deficits, particularly in patients with predisposing conditions. Early diagnosis through a combination of clinical features and neuroimaging remains crucial. Prompt management of the underlying causes, including hypertension and potential medication adjustments, is essential for optimizing patient outcomes.
